# Rituximab in Pemphigus Vulgaris: A Review of Monoclonal Antibody Therapy in Dermatology

**DOI:** 10.7759/cureus.40734

**Published:** 2023-06-21

**Authors:** Krishna Khandelwal, Vedika Jajoo, Kshitij Bajpai, Bhushan Madke, Roshan Prasad, Mayur B Wanjari, Pratiksha K Munjewar, Avinash B Taksande

**Affiliations:** 1 Medicine, Jawaharlal Nehru Medical College, Datta Meghe Institute of Higher Education and Research, Wardha, IND; 2 Internal Medicine, Jawaharlal Nehru Medical College, Datta Meghe Institute of Higher Education and Research, Wardha, IND; 3 Dermatology, Venereology and Leprosy, Jawaharlal Nehru Medical College, Datta Meghe Institute of Higher Education and Research, Wardha, IND; 4 Research and Development, Jawaharlal Nehru Medical College, Datta Meghe Institute of Higher Education and Research, Wardha, IND; 5 Medical Surgical Nursing, Srimati Radhikabai Meghe Memorial College of Nursing, Datta Meghe Institute of Higher Education and Research, Wardha, IND; 6 Physiology, Jawaharlal Nehru Medical College, Datta Meghe Institute of Higher Education and Research, Wardha, IND

**Keywords:** immunological mechanisms, efficacy, autoimmune disease, dermatology, rituximab, monoclonal antibodies, pemphigus vulgaris

## Abstract

Pemphigus vulgaris (PV) is a rare autoimmune blistering disorder that primarily affects the skin and mucous membranes. Conventional treatments for PV, such as corticosteroids and immunosuppressive agents, have limitations in terms of efficacy and long-term safety. Monoclonal antibody therapy, specifically rituximab, has emerged as a promising therapeutic approach in the management of PV. This review article provides a comprehensive overview of rituximab in the treatment of PV, with a focus on its efficacy, safety profile, and immunological mechanisms of action. The article begins with an introduction to PV and the significance of monoclonal antibody therapy in dermatology. It then explores the clinical presentation and underlying immune-mediated mechanisms of PV, highlighting the autoimmune nature of the disease. The rationale for using monoclonal antibody therapy, particularly rituximab, in PV is discussed, emphasizing the limitations of conventional treatments and the concept of targeted therapy. The review delves into the efficacy and safety of rituximab based on clinical studies, evaluating disease remission rates, duration, and relapse rates. Furthermore, the immunological effects of rituximab, including B-cell depletion and modulation of the immune response, are explored in detail. Comparisons between rituximab and conventional treatment modalities in PV are made, assessing clinical outcomes, safety profiles, and long-term efficacy. Challenges and considerations in rituximab therapy are discussed, including factors influencing its efficacy, optimal dosing, treatment duration, and the need for maintenance therapy.

## Introduction and background

Pemphigus vulgaris (PV) is a chronic autoimmune blistering disorder that affects the skin and mucous membranes. It is characterized by the formation of painful, flaccid blisters that can lead to significant morbidity and impaired quality of life for affected individuals. PV is considered a significant dermatological condition due to its potential for severe and potentially life-threatening complications, such as infection and fluid and electrolyte imbalances [1,2].

In recent years, monoclonal antibody (mAb) therapy has emerged as a promising treatment modality for various autoimmune diseases, including PV. mAbs are laboratory-produced molecules that target specific components of the immune system involved in disease pathogenesis. They offer a targeted and precise approach to treatment, minimizing systemic side effects often associated with conventional immunosuppressive agents [1,3].

One such mAb that has shown promise in the management of PV is rituximab. Rituximab specifically targets CD20-positive B cells, leading to their depletion and subsequent modulation of the immune response. By disrupting the B cell-mediated immune response, rituximab has demonstrated efficacy in achieving disease remission and reducing the need for long-term immunosuppressive therapy [1-3].

The objective of this review article was to provide a comprehensive overview of the use of rituximab in the treatment of PV within the field of dermatology. We will explore the background and significance of PV as a dermatological condition, discuss the potential of mAb therapy in autoimmune diseases, introduce the mechanism of action of rituximab, and outline the specific objectives of this review. Through this review, we aim to consolidate the current knowledge and evidence surrounding rituximab in the management of PV, evaluate its efficacy and safety profile, discuss its mechanisms of action, compare it with conventional treatment modalities, and identify future directions and emerging therapies in this field. By synthesizing the available literature and highlighting key findings, this review aims to provide clinicians and researchers with a comprehensive understanding of the role of rituximab in the treatment of PV and its implications for dermatological practice. It is hoped that this review will contribute to improving patient outcomes and guide further research in this evolving field.

## Review

Methodology

**Figure 1:**
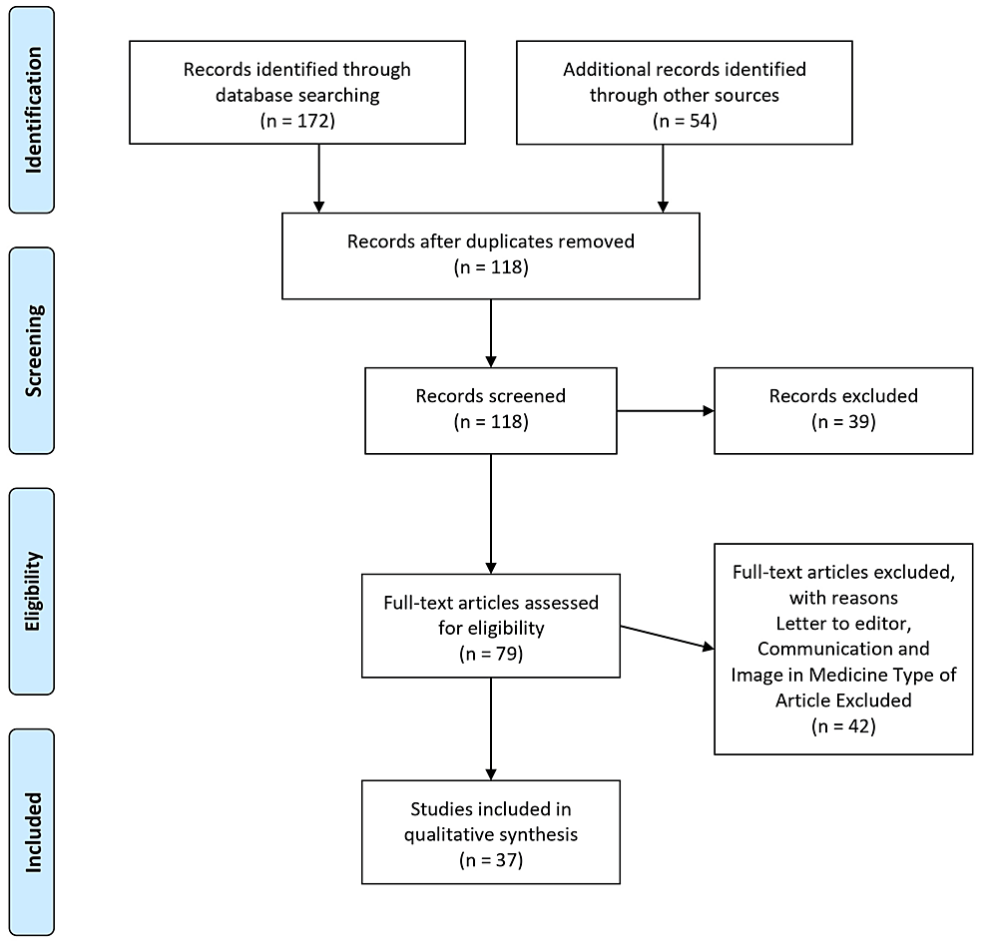
The selection process of articles used in this study. Adopted from the Preferred Reporting Items for Systematic Reviews and Meta-Analyses (PRISMA).

The review utilized a systematic approach to gather relevant literature. A comprehensive search of electronic databases, including PubMed, Embase, and Cochrane Library, was conducted to identify relevant studies. Additional sources, such as conference proceedings and gray literature, were also considered. The search strategy included keywords related to PV, rituximab, mAbs, dermatology, and autoimmune diseases. The search results were screened based on predetermined inclusion and exclusion criteria. Studies included in the review met the following criteria: (1) focused on the use of rituximab in the treatment of PV, (2) reported clinical outcomes, efficacy, safety, or immunological effects of rituximab, (3) published in peer-reviewed journals, and (4) available in the English language. Studies that did not meet these criteria were excluded. This rigorous selection process ensured that only relevant studies meeting specific criteria were included in the review, enhancing the validity and reliability of the findings. Figure 1 describes the selection process of articles used in our study.

Clinical presentation and pathogenesis of PV

PV is a rare autoimmune blistering disorder that primarily affects the skin and mucous membranes. It is characterized by the formation of intraepidermal blisters resulting from the loss of cell adhesion between keratinocytes, a process known as acantholysis. The blisters are fragile and easily rupture, leaving painful erosions and ulcerations [4,5].

The most common sites of involvement in PV are the oral mucosa, followed by the skin, scalp, genital mucosa, and other mucous membranes, such as the conjunctiva and esophagus. Oral involvement is almost universal in PV and often precedes cutaneous manifestations. The blisters and erosions in the oral cavity can cause significant pain and difficulty eating and speaking. Cutaneous involvement typically presents as flaccid blisters, which can occur in any body region, including the trunk, limbs, and scalp [4,5].

Mechanism of Action

PV is classified as an autoimmune disease resulting from an aberrant immune response against self-antigens. The primary target antigens in PV are desmoglein 1 (DSG1) and desmoglein 3 (DSG3), desmosomal cadherins that maintain cell-cell adhesion in the epidermis. Autoantibodies, known as pemphigus or pemphigus antibodies, are produced against these desmoglein proteins [6].

The immune-mediated mechanism in PV involves a breakdown in immune tolerance, producing autoantibodies against desmogleins. These autoantibodies bind to the extracellular domains of desmogleins, interfering with their adhesive function and causing acantholysis. The disruption of cell adhesion results in the formation of blisters and erosions in the affected tissues [7].

The exact triggers for the loss of immune tolerance in PV are not fully understood, but genetic predisposition and environmental factors likely play a role. Certain human leukocyte antigen (HLA) class II alleles, such as HLA-DR4 and HLA-DR6, have been associated with an increased risk of developing PV. In addition, factors such as viral infections, medications, and other autoimmune diseases may contribute to disease development or exacerbation [8,9].

The autoimmune nature of PV is further supported by circulating pemphigus autoantibodies in the serum of affected individuals. Immunofluorescence studies and enzyme-linked immunosorbent assays (ELISA) can detect these autoantibodies and aid in diagnosing PV. Understanding PV's clinical presentation and underlying immune-mediated mechanism is crucial for developing targeted therapies like rituximab. In the following sections, we will delve into the potential of mAb therapy in treating autoimmune diseases and explore rituximab's specific mechanism of action in PV [10].

The rationale for mAb therapy in PV

Conventional Treatment Modalities

The management of PV traditionally involves the use of systemic immunosuppressive agents, such as corticosteroids, immunosuppressive drugs (e.g., azathioprine, mycophenolate mofetil), and adjuvant therapies (e.g., intravenous immunoglobulins). While these treatments have shown efficacy in controlling disease activity, they are often associated with significant side effects and long-term complications [11].

Corticosteroids, such as prednisone, are the mainstay of PV treatment and are initially used in high doses to achieve disease control. However, their long-term use is limited by adverse effects, including weight gain, diabetes, osteoporosis, infections, and mood disturbances. Immunosuppressive drugs, on the other hand, have their own set of potential side effects, including myelosuppression, hepatotoxicity, and increased risk of malignancies. Additionally, many patients require prolonged treatment with these medications, making long-term compliance and monitoring challenging [2,8].

Targeted Therapy With mAbs

mAbs have revolutionized the treatment landscape in various autoimmune diseases, including PV. Unlike conventional immunosuppressive agents, mAbs offer a targeted approach by specifically binding to and modulating key components of the immune system involved in disease pathogenesis [12].

mAbs are engineered in the laboratory to recognize and bind to specific antigens, receptors, or cells. They can be designed to have various mechanisms of action, including blocking the function of a particular receptor, inducing antibody-dependent cellular cytotoxicity (ADCC), or directly targeting and depleting specific cell populations [13].

Mechanism of Action of Rituximab

Rituximab, a chimeric mAb, has emerged as a promising therapeutic option for PV. It specifically targets CD20-positive B cells, leading to their depletion and subsequent modulation of the immune response. CD20 is a surface protein expressed in pre-B cells, mature B cells, and some malignant B cells [14].

By binding to CD20, rituximab triggers ADCC, complement-mediated cytotoxicity, and apoptosis, resulting in the depletion of CD20-positive B cells. This depletion disrupts the production of pathogenic autoantibodies, including pemphigus autoantibodies, which are essential in the pathogenesis of PV [15].

Furthermore, rituximab has been shown to have additional immunomodulatory effects. It may alter the balance of T cell subsets, reduce the production of pro-inflammatory cytokines, and modulate the function of regulatory T cells, which helps restore immune tolerance and control autoimmune responses in PV. The targeted nature of rituximab therapy and its specific impact on B cells make it an attractive option for PV treatment. In the following sections, we will review the clinical efficacy and safety of rituximab in PV and explore the underlying mechanisms of action in greater detail [16,17].

Efficacy and safety of rituximab in PV

Clinical Studies

Numerous clinical studies and case series have investigated the efficacy of rituximab in treating PV. These studies have demonstrated promising results, highlighting the potential of rituximab as a therapeutic option for PV [18]. Clinical trials and retrospective studies have consistently shown that rituximab can induce disease remission in a significant proportion of PV patients who are resistant to or intolerant of conventional therapies. Rituximab has effectively reduced disease activity, improved clinical symptoms, and promoted re-epithelialization of skin and mucosal lesions [19].

The efficacy of rituximab in PV treatment has been assessed through various outcome measures, including disease remission rates, duration of response, and relapse rates. The findings from clinical studies suggest that rituximab can achieve disease remission in a substantial proportion of patients [20].

Remission rates vary among studies, with reported rates ranging from 60% to 90%. The duration of response following rituximab treatment can also vary, with some patients experiencing sustained remission for several months or even years. However, relapse rates have been observed, and long-term maintenance therapy may be required to prevent disease recurrence [21]. Factors influencing the response to rituximab therapy include disease severity, duration, and circulating pemphigus autoantibodies. Patients with milder disease and lower autoantibody levels tend to have better outcomes. Additionally, combination therapy with other immunosuppressive agents may be considered in certain cases to optimize treatment response.

Rituximab is generally well tolerated, with a favorable safety profile observed in PV patients. The most commonly reported adverse effects include infusion reactions, such as fever, chills, and skin rash, which can typically be managed with premedication and dose adjustment. Serious adverse events are rare but can include severe infections, infusion-related anaphylaxis, and reactivation of latent viral infections [5,11]. Long-term safety data for rituximab in PV are still evolving, and further studies are needed to assess its potential long-term risks, including the development of secondary malignancies. Close monitoring and appropriate pre-treatment evaluations are necessary to ensure patient safety [22].

Mechanisms of action and immunological effects of rituximab

Rituximab exerts its therapeutic effects in PV through multiple mechanisms, primarily targeting CD20-positive B cells. CD20 is a transmembrane protein expressed on the surface of pre-B cells, mature B cells, and some malignant B cells. Rituximab binds to CD20 and mediates B cell depletion through various mechanisms [23].

Rituximab triggers ADCC, wherein immune effector cells, such as natural killer (NK) cells, recognize rituximab-bound B cells and induce their destruction. This process leads to the depletion of circulating B cells and reduces the production of pathogenic autoantibodies [24]. Rituximab can induce complement-dependent cytotoxicity (CDC) by activating the classical complement pathway. Upon rituximab binding to CD20, complement proteins are recruited and activated, resulting in the lysis of targeted B cells [25].

Therapeutic effects of rituximab in PV

The therapeutic effects of rituximab in PV are not solely attributed to B-cell depletion. Additional immunological effects contribute to its efficacy in modulating the immune response and ameliorating disease activity.

Reduction in Pathogenic Autoantibodies

Rituximab specifically targets B cells responsible for producing autoantibodies against desmoglein proteins in PV. By depleting these B cells, rituximab effectively reduces the production of pathogenic autoantibodies, thus attenuating the immune response targeting desmogleins. This reduction in pathogenic autoantibodies improves disease activity and the healing of blistering lesions [26].

Modulation of T-Cell Responses

Rituximab treatment has influenced T-cell subsets and their cytokine profiles. It can reduce the number of CD4+ T helper cells, which is crucial in driving the immune response in PV. The decrease in CD4+ T helper cells leads to a diminished inflammatory response and a decrease in autoantibody production. Additionally, rituximab may promote the expansion of regulatory T cells (Tregs), which can help restore immune tolerance and control autoimmune responses. Tregs contribute to the suppression of autoimmunity by regulating the activity of other immune cells [27,28].

Impaired B Cell-T Cell Interactions

B cells have antigen-presenting capabilities and present autoantigens to T cells, stimulating the immune response. Rituximab-induced B-cell depletion disrupts these B cell-T cell interactions, leading to a decreased activation of T cells and dampening of the immune response. This disruption of B cell-T cell interactions further contributes to the suppression of autoantibody production and the attenuation of the immune response against desmoglein proteins [29].

Anti-Inflammatory Effects

Rituximab has been shown to reduce the production of pro-inflammatory cytokines, such as interleukin-6 (IL-6) and tumor necrosis factor-alpha (TNF-alpha). These cytokines play a crucial role in driving inflammation in PV. Rituximab exerts an anti-inflammatory effect by inhibiting the production of IL-6 and TNF-alpha, which ameliorates disease activity and inflammation in PV. This anti-inflammatory effect helps reduce the severity of blistering lesions and promotes healing [30]. Rituximab's multiple mechanisms of action and immunological effects in PV are essential for optimizing treatment strategies and predicting treatment responses. These mechanisms provide insights into potential targets for future therapeutic interventions in the management of PV.

Comparative analysis: rituximab vs. other treatment modalities

Rituximab was compared with the conventional immunosuppressive agents commonly used in treating PV. The aim is to evaluate rituximab's relative efficacy, safety profiles, and long-term outcomes compared to conventional modalities, such as corticosteroids and other immunosuppressive agents. Corticosteroids, such as prednisone, are the mainstay of PV treatment. They provide rapid disease control but are associated with numerous side effects and long-term complications. Rituximab has shown efficacy in achieving disease remission in corticosteroid-resistant cases, allowing for steroid dose reduction or even discontinuation [31]. Conventional immunosuppressive agents like azathioprine and mycophenolate mofetil (MMF) are often used as steroid-sparing agents in PV. While these medications can effectively control disease activity, they require long-term use and are associated with potential side effects, including myelosuppression and hepatotoxicity. On the other hand, rituximab offers an alternative option with a targeted mechanism of action and potentially lower long-term toxicity [11,19,20].

Prospective Commentary

Clinical outcomes: safety profiles and long-term efficacy are important factors when comparing rituximab with other treatment modalities in PV. Clinical studies have demonstrated that rituximab can induce disease remission in a significant proportion of PV patients who are resistant to or intolerant of conventional therapies. It has shown efficacy in reducing disease activity, improving clinical symptoms, and promoting re-epithelialization of skin and mucosal lesions. Comparative studies are needed to directly assess the comparative efficacy of rituximab versus conventional treatments [32].

Safety profiles: Rituximab is generally well tolerated, with infusion-related reactions being the most common adverse events. Serious adverse events, including severe infections and reactivation of latent viral infections, are rare but can occur. Conventional immunosuppressive agents have potential side effects depending on the specific medication used. Careful monitoring and individualized treatment decisions are crucial to optimizing safety profiles [33].

Long-term efficacy: The long-term efficacy of rituximab in PV is still being studied. While some patients experience sustained remission for months or even years, relapse rates have been observed, necessitating long-term maintenance therapy. Long-term follow-up data are needed to assess the durability of response and the need for retreatment or maintenance strategies. Comparative studies can provide insights into the long-term efficacy of rituximab compared to conventional treatments [34].

Challenges and considerations in rituximab therapy for PV

The efficacy of rituximab in PV can be influenced by various factors, including patient characteristics and disease severity.

Patient Characteristics

Age, gender, comorbidities, and immune status may affect the response to rituximab therapy. For example, older age and compromised immune function may impact the efficacy of rituximab. Additionally, concomitant autoimmune diseases or medications can influence treatment outcomes [11,35].

Disease Severity

The severity of PV during rituximab initiation can impact the response to therapy. Patients with more severe disease manifestations, extensive mucosal involvement, or high disease activity may require more aggressive treatment strategies, including higher doses or more frequent rituximab infusions [36].

Therapeutics

Dosing: The optimal dosing regimen for rituximab in PV is still under investigation. Different dosing strategies have been used in clinical practice, including the standard 375 mg/m² weekly for four weeks and the extended dosing regimen of 375 mg/m² weekly for four weeks, followed by additional infusions at 375 mg/m² every 3 or 6 months. Further research is needed to determine the most effective and individualized dosing regimen for different patient populations [37].

Treatment duration: The duration of rituximab therapy in PV is variable and depends on several factors, including treatment response, disease activity, and the occurrence of relapses. While some patients achieve sustained remission and may not require additional infusions, others may experience relapses or ongoing disease activity, necessitating long-term or maintenance therapy. Individualized treatment decisions should be based on patient response and disease status [7,15].

Maintenance therapy: The use of maintenance therapy following rituximab induction has been investigated to prevent relapses and maintain disease control. Various approaches have been proposed, including administering additional rituximab infusions on a scheduled basis or using other immunosuppressive agents in combination with rituximab. The optimal maintenance therapy strategy is still being defined, and further studies are needed to assess its efficacy and safety [14,26]. Other considerations in rituximab therapy include monitoring for treatment response and the need for retreatment, potential drug interactions, and cost-effectiveness.

## Conclusions

In conclusion, rituximab represents a significant advancement in the management of PV and holds promise for improving outcomes in dermatology. Further research, including comparative studies, long-term follow-up, and investigation of novel mAbs, is needed to optimize treatment strategies and maximize the potential impact of mAb therapy in dermatological practice. By harnessing the specificity and targeted mechanisms of action of mAbs, we can pave the way for more personalized and effective treatments in the field of dermatology.
